# Evaluation of the scale-up and implementation of mind, exercise, nutrition … do it! (MEND) in British Columbia: a hybrid trial type 3 evaluation

**DOI:** 10.1186/s12887-020-02297-1

**Published:** 2020-08-20

**Authors:** Sam Liu, Joy Weismiller, Karen Strange, Lisa Forster-Coull, Jennifer Bradbury, Tom Warshawski, Patti-Jean Naylor

**Affiliations:** 1 School of Exercise Science, Physical and Health Education, University of Victoria, Victoria, British Columbia Canada; 2 Juniper Consulting, Victoria, British Columbia Canada; 3 Childhood Obesity Foundation, Vancouver, British Columbia Canada

**Keywords:** Childhood obesity, Scale-up, Implementation

## Abstract

**Background:**

The Mind, Exercise, Nutrition … Do it! (MEND) childhood obesity intervention was implemented in British Columbia (B.C.), Canada from April 2013 to June 2017. The study objective was: a) to describe and explore program reach, attendance, satisfaction, acceptability, fidelity, and facilitators and challenges during scale-up and implementation of MEND in B.C. while b) monitoring program effectiveness in improving children’s body mass index (BMI) z-score, waist circumference, dietary and physical activity behaviours, and psychological well-being.

**Methods:**

This prospective, pragmatic implementation evaluation (Hybrid Type 3 design) recruited families with children and adolescents aged 7–13 with a BMI ≥ 85th percentile for age and sex. The 10-week MEND B.C. program was delivered in 27 sites, throughout all five B.C. health regions (Northern, Interior, Island, Fraser, and Vancouver Coastal) over 4 years. Families attended two weekly in-person group sessions aimed to increase physical activity and promote healthy eating. BMI z-score and waist circumference were measured at baseline and follow-up. Dietary and physical activity behaviours and psychological well-being were measured using validated questionnaires. A mixed-method approach was used to collect and analyze the data.

**Results:**

One hundred thirty-six MEND B.C. programs were delivered over 4 years. The program reached 987 eligible participants. 755 (76.5%) children and adolescents completed the program. The average program attendance was 81.5%. Parents reported the program content was easy to understand, culturally suitable, respectful of family’s financial situation, and provided adequate information to build a healthy lifestyle. Children achieved significant positive changes across all four evaluation years in BMI z-score (*d* = − 0.13), nutrition behaviours (*d* = 0.64), physical activity levels (*d* = 0.30), hours of screen time per week (*d* = − 0.38) and emotional distress (*d* = − 0.21). Challenges to continued program implementation included: recruitment, resource requirement for implementation, and the need to tailor the program locally to be more flexible and culturally relevant.

**Conclusions:**

The program reached a broad demographic of children and adolescents in B.C. Families were highly satisfied with the program delivery. MEND. B.C. at scale was effective across all four evaluation years in improving BMI z-score, lifestyle behaviours and psychological well-being among children. Future interventions need to explore strategies to enhance program delivery flexibility.

## Background

The prevalence of obesity has tripled in the last 15 years in Canada [1]. Over 25% of children have overweight or obesity in British Columbia (B.C.), Canada [1]. In many jurisdictions across B.C., families with children and adolescents (7–13 years of age) who are overweight have limited access to interventions that help to improve families’ lifestyle behaviours [2]. Obesity in childhood is also associated with a higher risk for poor psychosocial health, self-esteem and lower levels of self-confidence [2–4]. Consequently, preventing obesity in childhood has become a significant priority for public health agencies and health care providers. Thus, the B.C. Ministry of Health introduced the Mind, Exercise, Nutrition … Do it! (MEND) program from April 2013 to June 2017 to reduce this service gap. MEND B.C. is an evidence-based multi-component, healthy lifestyle intervention that includes behavioural, nutrition, and physical activity sessions delivered in community settings. MEND was extensively adapted for the Canadian population and was further localised for the B.C. population. The global MEND program is based on principles of nutritional and exercise science plus it draws from psychology, learning, and social cognitive theories and the study of therapeutic processes [5–7]. Previous randomized controlled studies as well as large-scale community-based trials have shown that the global MEND intervention significantly improves children’s body mass index (BMI) z-score, waist circumference, cardiovascular fitness, physical activity, and sedentary behaviours [5–9].

Family-based lifestyle interventions, such as MEND, are one of the principal approaches for achieving long-term weight control in children [2]. International recommendations coincide stating that the core elements of any intervention to address obesity in childhood should involve the whole family and include nutrition education, behaviour modification, and physical activity promotion [3, 10]. Encouraging the whole family to make behavioural changes decreases the focus placed on the overweight child’s dietary and physical activity behaviours and instead focuses on providing a supportive environment for making lifestyle modifications in the home setting [3, 7].

In order to achieve population-wide health improvement, these family-based childhood obesity intervention programs need to be ‘scaled-up’; extending the program reach [11]. Scaling up refers to the efforts to increase the impact of successfully tested health interventions to benefit more people and to foster policy and program development on a lasting basis [12–14]. Scaling up evidence-based childhood obesity interventions is a critical stage in translating evidence into practice. Currently, there are few scale-up studies in Canada and globally, particularly childhood obesity interventions using a Hybrid Type 3 design [15, 16]. Thus, the primary study objective was: a) to describe and explore program reach, attendance, satisfaction, acceptability, fidelity, and facilitators and challenges to implementation during scale-up and implementation of MEND B.C. while b) monitoring program effectiveness in improving children’s body mass index (BMI) z-score, waist circumference, dietary and physical activity behaviours, and psychological well-being.

## Methods

### Study design

The MEND B.C. scale-up and implementation project was a prospective, pragmatic implementation evaluation that used a Hybrid Type 3 evaluation design [17]. This type of design enables researchers to evaluate intervention implementation strategies while observing and gathering information on clinical intervention and related outcomes. MEND B.C. was a 10-week family-based childhood obesity intervention program for children and adolescents aged 7–13 with a BMI ≥ 85th percentile for age and sex, and their parents or caregivers living in B.C. MEND B.C. programs were delivered in 27 sites throughout all five B.C. health authority regions (Northern, Interior, Island, Fraser, and Vancouver Coastal) over 4 years (April 2013 to June 2017). The program demonstration phase took place in year 1 (April 2013 to June 2014) to establish program infrastructure and capacity before scale-up in years 2–4 (July 2014 to June 2017). Overall, 136 MEND B.C. programs were delivered over 4 years (Table 1). The study was approved by the University of Victoria Human Research Ethics Board (#13–117) and the University of British Columbia Children and Women’s Research Ethics Boards (#H13–01115).

**Table 1:** MEND B.C. programs delivered from April 2013 to June 2017

	Year 1 April 2013 – June 2014	Year 2 July 2014 – June 2015	Year 3 July 2015 – June 2016	Year 4 July 2016 – June 2017	Total
**Northern** **(3 sites)**	4	1	3	1	9
**Interior** **(6 sites)**	7	5	11	6	29
**Island** **(5 sites)**	8	8	7	5	28
**Fraser** **(9 sites)**	9	9	18	15	51
**Vancouver Coastal** **(4 sites)**	5	4	6	4	19
**Number of programs delivered per year**	33	27	45	31	136

### Participants

Children and adolescents were eligible to participate if they were between 7 and 13 years old, with a BMI ≥ 85th percentile for age and sex and had no contraindications for participating in physical activity or group sessions. MEND B.C. was a self-referral program. At least one parent or caregiver had to attend the sessions. Families were excluded if medical clearance was needed and not obtained for the child to participate in physical activity. Provincial and local recruitment strategies included advertisements in schools, community and recreation centers, libraries, general practitioners, pediatricians, local media, social media, word of mouth, and self-referrals. Each MEND B.C. program had the capacity to accommodate up to 15 families per program delivery cycle. Families may not repeat the program.

Stakeholders included MEND B.C. program delivery teams, which consisted of a programmer, theory leader, exercise leader and program assistant. In some cases, one individual performed more than one role e.g., a programmer who also served as a theory leader. Program-wide stakeholders included Childhood Obesity Foundation, provincial level delivery partners (British Columbia Recreation and Parks Association [BCRPA] and YMCA of Greater Vancouver), B.C. health authority regions (Northern, Interior, Island, Fraser, Vancouver Coastal) and localhost agencies that delivered MEND B.C. such as YMCA and BCRPA member recreation centres.

### Intervention: MEND

MEND was originally developed and extensively evaluated in the United Kingdom (U.K.) [5–7] and subsequently has been adapted and evaluated in Australia, the USA, Canada and the Netherlands. The MEND U.K. curriculum was thoroughly adapted to align with Canadian nutrition and physical activity guidelines [18, 19]. MEND B.C. was delivered in association with Healthy Weight Partnership, Inc. (HWP), the exclusive representative of MEND programs in North America (https://healthyweightpartnership.org/). The Childhood Obesity Foundation was funded by the Province of B.C. and licensed by HWP to establish, manage and deliver MEND in B.C. The Childhood Healthy Weights Intervention Initiative was considered to be a Demonstration Project in Year 1 (April 2013 to June 2014). At the end of the Demonstration phase responsibility for MEND operations and delivery was transferred to the Provincial Health Services Authority (PHSA) and implemented under the leadership of the Childhood Obesity Foundation in partnership with the Province of B.C.

MEND B.C. was offered for free to eligible families and delivered by trained leaders with recreation and health backgrounds. The programs ran for 10 weeks and were delivered throughout B.C. by local teams out of venues such as recreation centres. MEND included two weekly in-person group sessions (2 h per session; 20 sessions in total over the 10 weeks) that occurred on weekday evenings and weekends. Improving family’s knowledge, attitudes, social support and self-efficacy was the aim of the sessions. Sessions promoted behaviour change focusing on increasing physical activity, reducing sedentary behaviours and promoting healthy eating and used practical and engaging activities to deliver information. After program completion, participating families were given free three-month passes to their local recreation centres. Families were also given two-year access to “MEND World”, an online resource for maintaining and creating new healthy lifestyle changes after finishing the program.

The MEND B.C. scale-up could be described as a guided expansion of the same program to variety of different sties (or a horizontal scale-up strategy) [12]. The scale-up strategy was centrally driven, phased and involved multiple stakeholders in delivery of a standard intervention package supported by a dissemination approach that could be described as capacity-building [20]. A Provincial Stakeholder Advisory and a Management group were formed to guide dissemination and reflect the B.C. context in planning. MEND B.C. was managed by a MEND Provincial Manager and two Regional Coordinators (provided by stakeholders BCRPA and the YMCA of Greater Vancouver). Site level training was led by HWP and technical support was provided by the Regional Coordinators; including cross-site sharing meetings.

#### Outcomes

##### Reach

Program staff used feedback surveys at baseline to document the number of families enrolled, their demographic characteristics, and how families heard about MEND. Program staff also tracked the strategies used to promote programs in their communities, and the number of documented inquiries received that translated to enrollment.

##### Attendance

The program staff used a weekly survey to track the number of sessions each family attended and reasons for missing classes and dropping out. Drop-out was defined as a child who has attended < 5 of the 20 sessions inclusively. Attendance was calculated excluding children classified as drop-outs.

##### Fidelity

Weekly MEND sessions were assessed for delivery fidelity. At each site, the program staff indicated whether they were able to (yes/no) deliver all the required program content at each session from 2013 to 2016 (years 1 to 3). From 2016 to 2017 (year 4), the program staff used a five-point Likert scale to evaluate the quality of delivery (i.e. whether each lesson was delivered in a manner appropriate to achieving lesson objectives; 1 = very poor to 5 = very good) [21].

##### Program acceptability

Parents completed anonymous feedback surveys (Likert scale: 1 = not at all, and 5 = definitely) following the program to evaluate the following areas of program acceptability: whether the information given in the sessions was easy to understand, culturally suitable, respectful of the family’s financial situation, and adequate to build a healthy lifestyle.

##### Program satisfaction

Parents and children completed anonymous feedback surveys to assess whether they enjoyed attending the weekly sessions and learned about healthy living. Program satisfaction was measured using a five-point Likert scale (1 = not at all, and 5 = a lot or definitely). Opened-ended questions were used to identify particular aspects of the program that the families enjoyed.

##### Facilitators and challenges to implementation

The implementation facilitators and challenges were identified from stakeholder interviews (completed in the fall 2016 and winter 2017), leader feedback surveys (July 2014 through March 2017), document review (from July 2016 through March 2017), and participant feedback surveys (July 2014 through March 2017).

##### Effectiveness

Change in children’s BMI z-score (calculated based on the World Health Organization criteria) following the 10-week program [22]. Waist circumference was also measured using standardised procedures. Children’s cardiovascular fitness was measured using heart rate recovery 1 min after a validated 3-min step test [23]. Physical activity and sedentary behaviours were measured using the validated Physical Activity Questionnaire for Children (PAQ-C) and parent reports (hours of physical activity per week) [24]. Children’s dietary behaviours were evaluated using the MEND nutrition questionnaire [7]. This five-point Likert-scale questionnaire assessed the consumption frequency of sugar-sweetened drinks, whole grains, fast food, non-processed food, fruits and vegetables, family meals, and cooking from scratch. An overall dietary score was then computed. Children’s psychological well-being was evaluated by measuring emotional distress (Strength and Difficulties Questionnaires) [25].

#### Statistical analysis

A mixed-method approach was used to analyze the data. Descriptive statistics were used to summarize responses to the survey items to evaluate program reach, attendance, acceptability and satisfaction. Per-protocol analysis using paired t-tests compared mean changes in children’s BMI z-score, waist circumference, dietary and physical activity behaviours, and psychological well-being pre and post the MEND intervention. Effect sizes for each outcome variable were calculated using Cohen’s *d* (0.2 = small effect; 0.5 = medium effect; 0.8 = large effect). All quantitative data were analyzed using STATA version 13 (College Station, TX).

Open-ended questions from feedback surveys and stakeholder interviews were qualitatively analyzed for common themes. We used a framework analysis approach to analyze the content [26]. First, we used a coding system that was deductively developed based on a preliminary framework of categories generated by the evaluation team. We used this coding system to describe, sort, and analyze the interviewee’s quotes. Following the initial application of the themes, we modified the coding to include any missing themes that were not included in the initial scheme. Final presented themes were generated by integrating themes from all sources.

## Results

### Program reach

Recruitment activities led to a total of 987 eligible children and adolescents (7–13 years of age) participating in MEND B.C. over the 4 years. Most of the participants heard about MEND B.C. from posters and flyers (year 1: 29%; year 2: 52%; year 3: 48%; Year 4; 40%) and referrals (year 1: 13%; year 2: 12%; year 3: 25%; Year 4; 28%) were the most common source of recruitment. Other sources of recruitment included word of mouth (year 1: 5%; year 2: 12%; year 3: 9%; Year 4; 12%), Internet (year 1: 3%; year 2: 6%; year 3: 3%; Year 4; 5%), social media (year 1: 11%; year 2: 4%; year 3: 6%; Year 4; 4%).

Targeted recruitment activities were evaluated in-depth from September 2016 to January 2017 (during year 4). During this period, MEND B.C. information was delivered through 8900 engagements in the health sector, 2400 engagements in the physical activity and sport practitioner sector and 3500 engagements in the education sector.

During three program delivery cycles in year 4, program staff documented 415 participant families had contacted them. Out of those documented inquiries, at least 137 inquiries (33%) registered in a MEND B.C. program. Reasons for not registering in MEND B.C. included 1) not meeting the inclusion criteria (*n* = 52), schedule conflicts (*n* = 42), loss of interest or did not respond to follow-up calls or emails (*n* = 59). The participants recruited came from diverse educational, ethnic and socioeconomic backgrounds. Overall, the sample consisted of 48% male and 52% female (Table 2).

**Table 2:** Participant Demographic (*N* = 987)

	Year 1		Year 2		Year 3		Year 4	
	***n*** = 329		***n*** = 185		***n*** = 304		***n*** = 169	
**Child Gender**	**n**	**%**	**n**	**%**	**n**	**%**	**n**	**%**
Male	155	47.1%	93	50.3%	153	50.3%	76	45.0%
Females	174	52.9%	92	49.7%	151	49.7%	93	55.0%
**Child Ethnicity**								
Caucasian	180	57.3%	110	60.1%	153	52.6%	66	44.6%
First Nations	35	11.1%	15	8.2%	25	8.6%	16	10.8%
South and West Asians	26	8.3%	16	8.7%	27	9.3%	20	13.5%
Latin American	7	2.2%	4	2.2%	12	4.1%	5	3.4%
East and Southeast Asians	15	4.8%	12	6.6%	22	7.6%	11	7.4%
Mixed	41	13.1%	16	8.7%	36	12.4%	21	14.2%
Other (Arab, Black)	10	3.2%	10	5.5%	16	5.5%	9	6.1%
**Household Income**								
< $28,000	58	21.6%	32	19.8%	38	15.3%	34	25.6%
$28,000 - $40,999	41	15.3%	26	16.0%	50	20.2%	28	21.1%
$41,000 - $58,999	62	23.1%	32	19.8%	47	19.0%	21	15.8%
≥ $59,000	107	39.9%	72	44.4%	113	45.6%	50	37.6%
**Single Parent**								
Non-single parent family	216	70.6%	134	76.6%	196	71.0%	102	69.9%
Single parent family	90	29.4%	41	23.4%	80	29.0%	44	30.1%
**Parent Education**								
High school or less	82	27.7%	7	4.9%	7	3.3%	21	14.9%
Community college, trade school	123	41.6%	87	61.3%	126	59.7%	74	52.5%
University or above	91	30.7%	48	33.8%	78	37.0%	46	32.6%

Note: ^a^ Child ethnicity missing or undisclosed data: year 1: *n* = 15, year 2: = 2, year 3: *n* = 13, year 4: *n* = 21; ^b^ Household Income missing or undisclosed data: year 1: *n* = 61, year 2: = 23, year 3: *n* = 56, year 4: *n* = 36; ^c^ Single parent missing or undisclosed data: year 1: *n* = 23, year 2: = 10, year 3: *n* = 28, year 4: n = 23; ^d^ Parent education missing or undisclosed data: year 1: *n* = 33, year 2: = 30, year 3: *n* = 62, year 4: n = 28

### Program attendance

Seven hundred fifty-five (76.5%) participants completed the program. The average program attendance was 81.5%. The reasons for families not continuing the program include: not the right time for the family (19.3%), other priorities (11.7%), sickness (10.3%), change in family circumstance (10.3%), not the right program (4.8%), program too intensive (4.1%) Time not convenient (4.1%), and too difficult to get to (3.4%).

### Program Fidelity

The program staff rated whether they were able to deliver all the required program content at each session from 2013 to 2016 (years 1 to 3). The proportion of the sessions where all program content was delivered during year 1 (April 2014–June 2015), year 2 (July 2015–June 2016) and year 3 (July 2015–June 2016) were 93, 95 and 95%, respectively. During year 4 (June 2016–July 2017), when program staff used a Likert scale to evaluate the quality of delivery (1 = very poor to 5 = very good) 78% of all the lessons delivered were rated 4 or above and 21% had a rating of 3, with only 1% having a rating of 2.

### Program acceptability

Six hundred seventy-six parents completed the program acceptability survey over the 4-year assessment period. Program acceptability results are presented in Table 3. Over 90% of the parents surveyed post-program found the information to be easy to understand, culturally suitable for their family, respectful of their family’s financial situation, and provided adequate information to build a healthy lifestyle.

**Table 3:** Parents reported acceptability with the information provided by MEND B.C. (*n* = 676)

	Low to moderate levels of acceptability ^**a**^		High levels of acceptability ^**b**^	
	n	%	n	%
Adequate amount of information to help families build a healthy lifestyle	61	9.1%	615	90.9%
Respectful of family’s financial situation	48	7.1%	628	92.9%
Information was culturally suitable for the families	44	6.5%	632	93.5%
Information provided was easy to understand	30	4.5%	646	95.5%

Note: Includes eligible and non-eligible child participants as the surveys are anonymous. Therefore this 676 is not a sub-set of the 987 but of a more widely defined base. ^a^Low to moderate levels of acceptability group consists of combining values 1 and 3 combined - on a 5-point scale where 1 = “low”, 3 = “moderate” and 5 = “high satisfaction”. ^b^ high levels of acceptability group consists of combining values 4 and 5 combined - on a 5-point scale where 1 = “low”, 3 = “moderate” and 5 = “high acceptability”

### Program satisfaction

Six hundred seventy-six parents and 708 children and adolescents completed the program satisfaction survey. Program satisfaction results are presented in Table 4. Overall, the majority (> 80%) of the parents and the children found MEND B.C. satisfactory. Qualitative analysis of the parents’ feedback questionnaire revealed that they particularly enjoyed the following aspects of the weekly sessions: program content (e.g., MEND’s approach of combining psychology, behaviour change, exercise and nutrition content), program components (e.g., family physical activity sessions, specific sessions such as the grocery store tour, parent discussion sessions), program structure (e.g., group-format, family-based approach) and group dynamics (e.g., group discussion, group atmosphere, supportive, non-judgmental environment). Qualitative analysis of the children’s questionnaire revealed that the children particularly enjoyed some of the program components, specific sessions, interacting with the group facilitators, being with friends and making new friends.

**Table 4:** Families reported program satisfaction (Parents n = 676; Children *n* = 708)

	Low to moderate levels of satisfaction ^**a**^		High levels of satisfaction ^**b**^	
	n	%	n	%
Parents found the information was easy to act upon	80	11.9%	596	88%
Children enjoyed attending the weekly sessions	138	19.6%	570	80.4%
Children had fun interacting with the facilitators	54	7.6%	654	92.4%

Note: Includes eligible and non-eligible child participants as the surveys are anonymous. Therefore the 676 and 708 are not a sub-set of the 987 but of a more widely defined base (e.g., including siblings). ^a^ Low to moderate levels of satisfaction group consists of combining values 1 and 3 combined - on a 5-point scale where 1 = “low”, 3 = “moderate” and 5 = “high satisfaction”. ^b^ High levels of satisfaction group consists of combining values 4 and 5 combined - on a 5-point scale where 1 = “low”, 3 = “moderate” and 5 = “high satisfaction”

### Facilitators and challenges to implementation

The facilitators and challenges to implementing the program identified by stakeholders, program staff, and participants are summarized in Table 5.

**Table 5:** Implementation Facilitators and Challenges

**Facilitators**	
**Recruitment** • Promotions to families who have already identified their need for child weight management support, for example, those: o With children who have a BMI-for-age above the 97th percentile and/or having experienced a triggering situation or event o Talking with family physicians/pediatricians or going online to look for programming o Contacting their local recreation centres or providers to look for physical activity or nutritional programming – or going to events looking for information on these topics o Connecting with (former) MEND parents • Promotions to intermediaries in contact with multiple families with eligible children, for example, those: o Who are family physicians or pediatricians, in schools, in recreation centres or other physical activity or nutrition advice providers o By mail/email out, webinar, newsletter and/or at a conference • Promotions which use a multi-pronged and coordinated approach (at the neighbourhood/community, municipal and provincial levels) are well-branded, use key messages which resonate well, are boosted by champions, are ongoing, are synchronized with other schedules (e.g., for newsletters) and are sufficiently funded. • Using a combination of promotions which are more widespread, though with lower levels of conversions (e.g., posters/flyers and social media), and promotions which have a narrower spread but have higher levels of conversion (e.g., referrals). • Program delivery elements which encourage recruitment include – program content, ability to meet eligibility criteria (e.g., age, BMI and/or risk factors criteria), convenience of location, free (no cost), timing/schedule, inclusion of siblings. • Knowing your communities - what works in one community may not work at all in another.	
**Delivery** • An overall approach which combines nutrition, exercise and psychology. One which is group-based (providing discussion, support, interpersonal connections/friendships and culturally diverse). One which is family-based – involving parents (or other caregivers) as well as children. • Highly qualified, skilled, motivated, enthusiastic, well-prepared staff with strong community connections. Staff continuity - enabled where the organizational staffing structure is not based on short-term contracts. Strong centralized training, responsive external support for staff (i.e. Regional Coordinators) and the sharing of resources among facilitators/teams. • Program sessions on nutrition and healthy eating as well as engaging physical activity sessions, especially games. Activities which are interactive, hands on, (age-) appropriate and fun. • Delivery elements and logistics such as good venue facilities and spaces. Accessible session times and program lengths. Establishing and communicating clear expectations around behaviour. Using specific retention strategies (emails between sessions, follow up with families with poor attendance, promote future sessions in current sessions, fun/engaging sessions). Having committed/engaged families.	
**Outcomes** • Short-term outcomes: o families that are satisfied with the program and making lifestyle changes while they are in it o statistically significant positive changes in measures consistently achieved across all four evaluation time periods	
**Challenges**	
Recruitment • Connecting with communities when there is: o Lack of community size/awareness/interest and/or o Lack of program staff time and/or available promotional materials • The BMI eligibility criterion and the challenges faced by delivery team staff and partners around what language to use when speaking with parents about their child’s weight • Program delivery elements (twice a week, inconvenient locations or session times) • Lack of clear and direct communication with sites about provincial level recruitment activities so that site staff are aware of these activities and can leverage them through complementary local promotions.	
**Delivery** • A disconnect in the overall approach among stakeholders as to whether the program should be treated as a medical intervention program or a community healthy living program. This results in some confusion and communication challenges between partners, and can contribute to difficulty recruiting participants. • The effects of programs not running – on facility bookings, staff turnover, smaller group dynamics (as a result of low attendance). • Within programs – participant behavioural issues, broad age groupings, the strong facilitation skills required, content issues (e.g., weight-focused language, multicultural content needs, lack of cultural relevance for First Nation families, recent/updated nutrition content needs and/or more time on physical activity relative to classroom time), twice a week delivery (rather than once a week delivery) and data collection issues (missing or un-entered data and the high number of questionnaires).	
**Outcome** • Long-term outcomes – the lack of follow up or maintenance activities means nothing is in place after the program. Thus, there is no way to support changes over time and/or to confirm long- term impacts e.g., the extent to which recreation passes are being used, whether changes made continue or whether new changes are being made.	

### Program effectiveness

The effectiveness of MEND in changing anthropometric, lifestyle behaviour and psychological outcomes are shown in Table 6. The percentage of children and adolescents that were obese (BMI-for-age was above the 97th percentile) decreased from 82% at baseline to 79% at follow-up. The percentage of children and adolescents that were classified as overweight (BMI-for-age was between 85th and 97th percentile) following the intervention remained at a similar level as the baseline (18%). However, 3% of the children and adolescents reached BMI-for-age below the 85th percentile following the intervention. Children and adolescents also achieved significant positive changes across all four evaluation periods in nutrition behaviours (*d* = 0.64), physical activity (*d* = 0.40), hours of screen time per week (*d* = − 0.38) and emotional distress (*d* = − 0.21). Cardiovascular fitness, measured by recovery heart rate, significantly improved in year two (*d* = − 0.22) and year four(*d* = − 0.23).

**Table 6:** MEND B.C. Program Effectiveness Outcomes

	Year 1				Year 2				Year 3				Year 4				Overall			
	Baseline Mean (SD)	Follow-up Mean (SD)	Δ (95CI)	ES	Baseline Mean (SD)	Follow-up Mean (SD)	Δ (95CI)	ES	Baseline Mean (SD)	Follow-up Mean (SD)	Δ (95CI)	ES	Baseline Mean (SD)	Follow-up Mean (SD)	Δ (95CI)	ES	Baseline Mean (SD)	Follow-up Mean (SD)	Δ (95CI)	ES
BMI z-score	2.82 (0.85)	2.71 (0.84)	−0.11* (− 0.13, − 0.08)	− 0.13	3.02 (1.12)	2.85 (1.06)	− 0.16* (− 0.19, − 0.13)	− 0.16	2.55 (0.95)	2.43 (0.95)	− 0.12* (− 0.14, − 0.09)	− 0.13	2.65 (0.9)	2.52 (0.93)	−0.12* (− 0.15, − 0.09)	−0.14	2.74 (0.96)	2.62 (0.95)	−0.12* (− 0.14,-0.11)	−0.13
Waist circumference (cm)	89.5 (0.8)	88.6 (11.8)	−0.9* (− 1.3, − 0.5)	−0.11	89.4 (1)	88.2 (11.5)	−1.2* (− 1.7, − 0.7)	−0.15	83.7 (0.8)	83.1 (13)	−0.5* (− 1, 0)	− 0.07	84.9 (11.6)	84.5 (11.9)	− 0.4 (− 1, 0.2)	−0.03	86.9 (0.5)	86.1 (12.4)	−0.7* (− 1,-0.5)	−0.09
Nutrition score (0–28)	17.6 (4.3)	21.7 (6.9)	4.1* (3.5, 4.6)	0.71	18.7 (4.4)	22.1 (6.4)	3.4* (2.8, 4)	0.62	18 (4.1)	21.1 (6.6)	3.1 * (2.7, 3.6)	0.56	17.8 (3.7)	21 (5.6)	3.3 *(2.6, 4)	0.67	18.0 (4.2)	21.5 (6.5)	3.5* (3.2,3.8)	0.64
Physical activity (hours/week)	10.4 (6.2)	14.1 (6.9)	3.7* (2.8, 4.7)	0.56	10.3 (5.9)	13.2 (8.2)	2.9* (1.7, 4.2)	0.41	11.2 (5.7)	13.1 (7.2)	1.9* (1, 2.7)	0.29	11.5 (6.4)	12.9 (5.7)	1.4 (0.3, 2.5)	0.23	10.8 (6)	13.4 (7.1)	2.6* (2.1,3.1)	0.40
PAQ-C [1–5]	2.67 (0.63)	2.95 (0.75)	0.27* (0.19, 0.35)	0.40	2.76 (0.63)	2.89 (0.69)	0.13* (0.03, 0.23)	0.20	2.71 (0.61)	2.88 (0.62)	0.18* (0.10, 0.25)	0.28	2.72 (0.7)	2.91 (0.67)	0.18* (0.08, 0.29)	0.28	2.71 (0.64)	2.91 (0.69)	0.20* (0.16, 0.25)	0.30
Recovery heart rate (beats/minute)	105.1 (18.3)	103.1 (17.5)	−2 (−4.3, 0.3)	−0.11	109.8 (18.1)	105.8 (18.8)	−3.9* (−6.4, −1.5)	−0.22	101.5 (19.1)	101.3 (16.8)	−0.2 (− 2.5, 2)	− 0.01	104.2 (19.4)	100.3 (14.7)	− 3.9* (− 6.6, − 1.3)	−0.23	104.6 (18.9)	102.5 (17.2)	−2.1* (− 3.3,-0.9)	− 0.12
Hours screen time/week	13.7 (9)	9.8 (6.9)	−3.9* (−5, − 2.8)	− 0.49	12 (9)	9.2 (8.2)	−2.7* (− 4.6, − 0.8)	−0.33	11 (7.3)	9.6 (7.2)	− 1.5* (− 2.5, − 0.5)	−0.19	12.9 (8.6)	8.6 (5.7)	−4.3* (− 5.8, − 2.8)	− 0.59	12.4 (8.5)	9.4 (7.1)	−3.0* (− 3.6,-2.3)	−0.38
Emotional Distress (0–40)	11.3 (6.2)	9.7 (6.2)	−1.6* (− 2.3, − 1)	− 0.26	10.7 (5.9)	9.2 (5.9)	−1.5* (− 2.4, − 0.7)	−0.25	11.5 (6)	10.5 (6.1)	−1.0* (− 1.5, − 0.4)	−0.17	11.1 (5.8)	10.1 (5.1)	−1 (− 1.8, − 0.3)	−0.18	11.2 (6)	9.9 (6.1)	−1.3* (− 1.6,-1)	− 0.21

Note: Δ = change in outcomes at follow-up relative to baseline; *ES* effect size, *SD* Standard Deviation, *CI* Confidence intervals. *p < 0.05

## Discussion

This study aimed to evaluate the implementation and scale-up of MEND in B.C., Canada. Scale-up of family-based childhood obesity intervention programs is likely a key component of strategies to combat the upward trends in childhood obesity [11]. MEND B.C. was an example of how a cross-sectoral partnership can work together to implement and scale-up a comprehensive family-based behavioural health program over 4 years. The program reached a broad demographic of children and adolescents (7–13 years of age) in B.C. At scale, MEND B.C. was effective in improving BMI z-score, lifestyle behaviours and psychological well-being among children. However, we also identified several challenges to continuing the implementation of MEND, which included in B.C., targeting a program to overweight and obese children, program resource requirements, stakeholder need for more program delivery flexibility and tailoring the program to indigenous and non-traditional families.

Scaling up efficacious interventions into real-world settings is critical to prevent delays in community access to effective health services [14]. The method of MEND B.C. implementation and scale-up followed the best-practices and strategies identified to support the scale-up of public health initiatives [12, 14]. The main strategies that supported the successful MEND B.C. scale-up were active engagement with multilevel stakeholders (e.g., B. C Ministry of Health, Childhood Obesity foundation) and delivery agents: YMCA of Greater Vancouver and BCRPA, PHSA) to implement and evaluate the program and to enable the stakeholders to work together to tailor the scale-up approach to the B.C. context. Other facilitators included: highly qualified and motivated staff with strong community connections, staff continuity, highly responsive external support (Regional Coordinators) and strong centralized training.

Our recruitment strategies enabled our team to reach families that were characteristic of British Columbian families. According to a recent census of all B.C. families with children at home, 27% were single-parent families [27], which was similar to participating MEND B.C. families. Similarly, according to the latest National Household Survey, 27% of British Columbians were members of visible minorities [28], which was comparable to MEND B.C. families. Participating MEND families of Aboriginal identity were more represented in the program than amongst the general B.C. population. According to the National Household Survey’s B.C. population subset, 5 % were of Aboriginal identity, lower than MEND B.C. families [29]. Overall, our successful recruitment effort could be attributed to the wide variety of strategies to raise awareness about MEND B.C. in their communities, and each site’s recruitment strategy was tailored to its community.

The effectiveness of the MEND B.C. scale-up trial in improving BMI z-score and lifestyle behaviours were similar to the previous MEND studies [5–9, 30]. Improvements in nutrition and physical activity behaviours for children and adolescents had a larger effect than changes in BMI z-scores. The magnitude of change in BMI z-score was slightly smaller compared with the MEND U.K. randomized control trial [7]. A previous MEND program delivered in community settings in the United Kingdom also reported a smaller magnitude of improvements in children’s BMI z-score relative to the MEND randomized controlled trial [7]. This has been described as the ‘scale-up penalty’; with scaled-up childhood obesity interventions in a recent systematic review achieving 75% or less than original efficacy studies [31]. The reduced magnitude of physiological and lifestyle behaviour may also be attributed to the longer follow-up time in the randomized controlled trial (24 weeks vs. 10 weeks in this study). Although our primary outcome (BMI z-score) significantly reduced in all program delivery years, some secondary outcomes (e.g. sedentary behaviours, psychosocial outcomes) were not significantly changed in some of the program delivery years. The lack of improvement in these outcomes in some program years may be attributed to sample size, group dynamics or wider environmental factors that impair successful weight management. A longer intervention duration may be required to observe a greater change in these outcomes [32].

The overall family-based approach used with MEND was perceived as positive by those families that participated. However, after 4 years provincial stakeholders suggested that several important program challenges needed to be addressed in order for them to sustain delivery of the program. First, they identified a need to address the disconnect among stakeholders as to whether the program should be treated as a targeted intervention program for overweight or obese children or a more inclusive community-based family healthy living program without BMI restrictions (>85th percentile). With the BMI criteria in place, program staff and partners faced challenges around what language to use when speaking with parents about their child’s weight. This contributed to recruitment challenges. Second, that future programs needed to incorporate cultural and determinants of health lenses in order to enhance program relevance to population subgroups such as Indigenous families and those experiencing the impacts of poverty; and reduce deterrents for these subgroups participating (e.g. moving beyond free programming). Third, stakeholders wanted greater local program delivery flexibility and had concerns about the resources needed to run the program.

With the advancement in Internet-enabled digital devices (e.g., smartphones, tablets, computers, wearables) and improved access to the Internet, there is emerging evidence these innovative digital technologies can help deliver population-based chronic disease prevention programs without overtaxing health-care resources [33–35]. Future studies need to examine the effectiveness, implementation and scale-up of these Internet-based interventions aimed to manage childhood obesity.

There were several limitations to this study. First, as a Type Three hybrid design the primary focus was on evaluating implementation with a secondary objective of monitoring program outcomes [17], thus there was no control group. Second, there were no follow up measurements beyond the10-weeks program duration. Therefore, it is unknown if changes observed and reported during the program were maintained after families completed MEND B.C. Third, there was a selection bias in our sample. The majority of our sample (82%) consisted of children and adolescents with BMI-for-age above the 97th percentile and therefore, may affect the generalizability of our findings beyond this population group. Fourth, families who withdrew did not complete program acceptance and satisfaction feedback forms at the end of the program. Some families who did not meet the study inclusion criteria also completed the MEND B.C. program, and their responses were included in the program feedback survey. It is impossible to know if families that withdrew or families that did not meet the study inclusion criteria would have responded differently than families that were retained. All delivery sites are located in urban areas; thus, the generalizability of our finding to rural areas is limited. Finally, a cost-effectiveness analysis was not conducted in this study. Future studies need to conduct an economic evaluation to determine the feasibility of implementing such intervention in other countries.

## Conclusion

MEND B.C. was successfully scaled-up and implemented in B.C., Canada from 2013 to 2017. The scale-up initiative was founded on cross-sectoral partnerships and reached communities and families across British Columbia; with 987 overweight (18%) and obese (82%) children and adolescents (7–13 years of age) and their families provided with extensive lifestyle intervention support. MEND. B.C. ‘at scale’ remained effective in improving BMI z-score, lifestyle behaviours and psychological well-being among children. Families were highly satisfied with the program delivery and found the program met their needs. However, recruitment was challenging over all 4 years of implementation. In addition, the resource requirement for implementation and the need to tailor the program locally to be more flexible and culturally relevant for B.C. were challenges to continued program implementation.

## Data Availability

The datasets used and/or analysed during the current study are available from the corresponding author on reasonable request.

## Abbreviations

B.C.: British Columbia
BMI: Body Mass Index
MEND: Mind, Exercise, Nutrition … Do It!
PAQ-C: Physical Activity Questionnaire for Children
